# Development and validation of a novel approach for quantifying dimensions of the lateral ligaments in human ankle dissections

**DOI:** 10.1111/joa.14267

**Published:** 2025-05-14

**Authors:** Sophie J. Mok, A. Hamish R. W. Simpson, Jennifer Z. Paxton

**Affiliations:** ^1^ Anatomy@Edinburgh, Edinburgh Medical School: Biomedical Sciences University of Edinburgh Edinburgh UK; ^2^ Centre for Discovery Brain Sciences, Edinburgh Medical School: Biomedical Sciences University of Edinburgh Edinburgh UK; ^3^ Centre for Inflammation Research University of Edinburgh Edinburgh UK

**Keywords:** ATFL, CFL, dimension methodology, lateral ligaments of the ankle, ligament dimensions, morphometrics, PTFL

## Abstract

The lateral ankle ligaments, composed of the anterior talofibular (ATFL), calcaneofibular (CFL), and posterior talofibular ligaments (PTFL) are frequently subject to injury. While conservative and surgical treatment methods have had some positive outcomes, high rates of re‐injury, chronic ankle instability, and pain remain, prompting the investigation of tissue‐engineered applications in the treatment of lateral ligament injuries. In order for tissue‐engineered construct design to be undertaken, a complete understanding of the native anatomy of the lateral ankle ligaments must be obtained. To date, substantial data exist on the anatomical structure of the lateral ankle ligaments, particularly surrounding their dimensions throughout movements of the ankle complex. Despite this, current literature does not consider the dynamic nature of the lateral ankle ligaments when assessing true ligament length nor the ability of ligaments to stretch and recoil throughout joint movement. Existing methodologies for measuring the lateral ankle ligaments commonly use instruments with limited flexibility and simply measure the distance between attachment points, not accounting for any degree of relaxation within the ligament. Therefore, this study aims to establish a new methodology that considers the curvatures and form of the lateral ankle ligaments throughout all movements of the ankle complex. Cadaveric dissection was performed on 21 ankles to fully expose the lateral ankle ligaments. The full length of the ATFL, CFL, and PTFL was measured following a newly developed ‘String for Dynamic Tissue’ (SDT) method. Flax‐coated wax string was aligned and moulded to the surface and curves of each ligament from the most proximal attachment point to the most distal attachment point, along the same plane and cut to size. Measurements for each ligament were assessed throughout all degrees of movement of the ankle complex: plantarflexion, dorsiflexion, inversion, eversion, and neutral. A digital calliper was used to measure the exact string length, representing both the relaxed and taut ligament's full ligament length. Across all samples, the full length of the ATFL ranged between 21.20 and 33.80 mm (*n* = 20) throughout all movements of the ankle complex. Measurements of the CFL ranged between 28.66 and 44.44 mm (*n* = 21), while full‐length measurements of the PTFL ranged from 27.90 to 39.80 mm (*n* = 21). Ligament dimensions of the relaxed ligament were greater when compared to the current literature, while dimensions of the taut ligament closely resembled data currently available. The SDT method not only enables accurate measurement and assessment of non‐linear structures but also highlights the importance of considering complete structural form, emphasizing the need to move beyond merely measuring the linear distance between two points. This method will have multiple applications within the anatomical and biomechanical fields and across a range of tissue types and locations.

## Graphical abstract

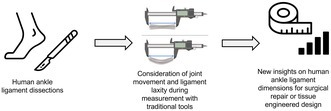

Traditional measurement techniques in cadaveric dissection fail to account for the changes in soft tissue length during joint movement. We have developed a new, simple method to obtain more accurate measurements of ligament length and validated using the lateral ankle ligaments group.

## 1 INTRODUCTION

The lateral ligaments of the ankle, composed of the anterior talofibular (ATFL), calcaneofibular (CFL) and posterior talofibular ligaments (PTFL), constitute 85% of all ankle injuries and account for 16%–40% of sport‐related injuries (Beynnon et al., 2002; Delahunt et al., 2018; Ferran & Maffulli, 2006; Gribble et al., 2016; Halabchi & Hassabi, 2020; Li et al., 2019). Furthermore, lateral ligament injuries represent a substantial portion, ~ 5%, of all hospital emergency cases, amounting to an average of 5600 lateral ankle injuries per day (Bestwick‐Stevenson et al., 2021; Bleakley et al., 2010; Cooke et al., 2003; Doherty et al., 2014; Lamb et al., 2005, 2009; Palmer‐Green et al., 2016), posing a significant burden within the national healthcare system.

The ATFL is widely recognized as the most vulnerable component of the lateral ligamentous complex, playing a significant role in the majority (around 85%) of ankle injuries and is frequently involved in isolated injuries separate from the remaining lateral ligaments (D'Hooghe et al., 2020; Casado‐Hernández et al., 2021). Conversely, isolated injuries to the CFL and PTFL are relatively rare and typically accompany injury to the ATFL (Furdson & Platt, 2012; Le & Tiu, 2023). Involvement of the CFL ranges from ~20% to 75% of cases, whereas the PTFL is implicated in <15% of lateral ankle injuries (Casado‐Hernández et al., 2021; Le & Tiu, 2023; Li et al., 2019; Peng et al., 2023; Swenson et al., 2013).

Following injury to a ligament, natural physiological healing begins to take place. However, as a result of poor vascular perfusion and low cellular count, the healing properties of most ligaments, particularly at the enthesis, are poor (Kwansa & Freeman, 2015; Yilgor et al., 2012). This leads to weak and disorganized tissue repair, prone to re‐injury (Kwansa & Freeman, 2015, Yilgor et al., 2012). Initial treatment incorporates the PRICE protocol (protection, rest, ice, compression, elevation), coupled with early mobilization with support and strengthening exercises (Thompson et al., 2017). Surgical intervention is usually only necessary in advanced ligament injury and rupture (Petersen et al., 2013). While conservative and surgical treatment methods have achieved positive healing outcomes, the risk of re‐injury and reports of residual pain remain high (Herzog et al., 2019).

Approximately 45% of patients experience incomplete recovery within a 3‐year period following injury (Feger et al., 2017; van Rijn et al., 2008). Furthermore, the occurrence of re‐sprain/re‐injury and recurrent symptomatic chronic ankle instability following an acute ankle sprain has been documented in 10%–40% of cases, with residual symptoms persisting in up to 40% of individuals (Al‐Mohrej & Al‐Kenani, 2016; Delahunt et al., 2018; Doherty et al., 2014; Renström & Lynch, 1998; Sarcon et al., 2019; Takao & Glazebrook, 2022; van Rijn et al., 2008; Zhang et al., 2023). In a study conducted by Kemler et al. (2016), it was revealed that following an inversion sprain, 18% of patients exhibited repetitive re‐injury due to chronic ankle instability. Additionally, 45.5% of patients continued to report ankle pain during examination, 25% presented with clinical symptoms indicative of anterior ankle impingement, and 82% displayed osteophyte bone formation, all within a span of 2–5 years following the initial injury (Kemler et al., 2016). At present, there are more than 50 surgical approaches in the treatment and reconstruction of the lateral ligaments of the ankle, thereby suggesting that a fully comprehensive and satisfactory method has yet to be identified (Becker et al., 1994, 1996; Bohnsack et al., 2002; Khawaji, 2016; Peters et al., 1991; Sammarco, 2001). Poor treatment outcomes, high rates of re‐injury, and chronic instability have led to the exploration of tissue engineering in the management and treatment of ligament injuries.

Given the significant incidence of injury affecting this group of ligaments, extensive research has been conducted, detailing their macroscopic structure, dimensions, and histological arrangement. However, despite the wealth of available data, notable gaps in knowledge remain, specifically related to the ligament dimensions. Gaining a comprehensive understanding of the dimensions of the lateral ankle ligaments is crucial in unlocking valuable insights for their effective repair in response to injury. Specifically, the dimensions serve as a guide for the precise positioning of bony tunnels or in ligament length adjustment to improve laxity and stability during surgical intervention (Jorge et al., 2018). Furthermore, an understanding of dimension variation offers insights into the mechanisms of injury, aiding in the development of appropriate management strategies. Finally, obtaining data on ligament dimensions is crucial to achieve optimal efficacy in tissue‐engineered design, as a thorough knowledge of the native anatomy enables accurate replication in artificial tissue design (Loukopoulou et al., 2022).

The range of dimensions of the lateral ligaments of the ankle, particularly ATFL, has been reported previously, resulting in a database of measurements (Table 1). While all dimension measurements naturally exhibit a range of results, current full‐length dimensions for the lateral ligaments of the ankle display considerable variability, with some ligaments measuring more than three times previously reported values (Table 1). Naturally, human variation (foot size and shape, variations in fascicular structure, bone size, sex etc.) accounts for a portion of this variety; however, such a large discrepancy between measurements suggests inconsistencies in the methodology used to assess ligament dimensions.

**TABLE 1:** Dimensions of the lateral ankle ligaments as reported in previous studies (±SD), with values in brackets indicating the range reported in the literature.

Authors	Lig.	Lengths (mm)	Width (mm)	Thickness (mm)	Ankle position	Study type
Ahmed et al. (2024)	ATFL CFL PTFL	14.24 ± 2.40 (12.00–25.00) 18.40 ± 3.90 (10.40–28.70) 20.90 ± 3.30 (14.40–28.60)	7.60 ± 2.00 (4.20–10.70) 5.20 ± 1.30 (3.20–8.00) 6.20 ± 1.40 (4.30–9.10)	– – –	N	CD
Bai et al. (2013)	ATFL CFL PTFL	20.08 ± 2.16 (17.92–22.96) 32.72 ± 9.17 (28.31–41.82) –	8.75 ± 1.80–9.26 ± 1.34 4.76 ± 0.62–5.08 ± 0.77 –	– – –	NI	CD
Burks and Morgan (1994)	ATFL CFL PTFL	20.00–24.80 35.80 –	4.60–7.20 5.30 –	– – –	NI	CD
Buzzi et al. (1993)	ATFL CFL PTFL	17.50 (15.00–22.00) 20.00–30.00 16.00–25.00	10.80 (8.00–13.00) 5.00–11.00 –	– – –	ALL	CD
Clanton et al. (2014)	ATFL CFL PTFL	14.70–16.40 24.70 –	– – –	– – –	N	CD
Croy et al. (2012)	ATFL CFL PTFL	18.60 ± 1.50 (17.10–20.10) – –	– – –	– – –	P,D,I,N	US
de Asla et al. (2009)	ATFL CFL PTFL	11.00–23.50 24.00–30.90 –	– – –	– – –	ALL	MRI
Dimmick et al. (2008)	ATFL CFL PTFL	– – –	– – –	2.19 ± 0.6 2.13 ± 0.5 –	N	MRI
Edama et al. (2018)	ATFL CFL PTFL	14.90–27.50 – –	3.80 ± 1.60–7.50 ± 2.60 – –	– – –	N	CD
Hong et al. (2022)	ATFL CFL PTFL	13.20–18.40 – –	2.60–4.0 – –	– – –	N	MRI
Inchai et al. (2021)	ATFL CFL PTFL	– – 26.11 ± 3.11 (23.00–29.22)	– – 7.65 ± 1.15	– – 3.43 ± 0.52	NI	CD
Inchai et al. (2023)	ATFL CFL PTFL	11.96–22.50 34.67 ± 7.27 (27.40–41.94) 27.23 ± 3.35 (23.88–30.58)	3.96 ± 1.11–8.04 ± 1.66 5.08 ± 1.10 8.00 ± 1.27	0.55 ± 0.22–1.06 ± 0.32 1.11 ± 0.37 1.84 ± 0.51	NI	CD
Kawabata et al. (2023)	ATFL CFL PTFL	19.98–28.06 – –	– – –	– – –	P	US
Khawaji (2016)	ATFL CFL PTFL	19.58 ± 3.47 (16.11–23.05) 30.18 ± 5.03 (25.15–35.21) 24.03	2.17 ± 0.78–6.02 ± 1.64 4.19 ± 1.55 5.52	0.94 ± 0.35 1.40 ± 0.48 2.06	ALL	CD
Khawaji and Soames (2015)	ATFL CFL PTFL	18.81–21.06 – –	2.17 ± 0.78–6.02 ± 1.64 – –	1.01 ± 0.35 – –	ALL	CD
Kitsoulis et al. (2011)	ATFL CFL PTFL	– 31.83 –	– 4.42 –	– 1.58 –	P,D,I,N	CD
Kobayashi et al. (2020)	ATFL CFL PTFL	11.80–27.40 21.70–31.50 22.00–30.60	2.40–10.70 3.50–7.50 4.30–8.00	– – –	NI	CD
Kristen et al. (2019)	ATFL CFL PTFL	12.81–18.12 – –	– – –	– – –	ALL	US
Kwon et al. (2019)	ATFL CFL PTFL	20.60 ± 2.40 (18.20–23.00) – –	9.40–17.10 – –	– – –	N	CD
Liu et al. (2017)	ATFL CFL PTFL	12.40–18.80 – –	2.80 ± 0.70 (2.10–3.50) – –	– – –	N	MRI
Luo et al. (1997)	ATFL CFL PTFL	11.50 ± 0.25 (11.25–11.75) 20.60 ± 0.29 (20.31–20.89) 14.20 ± 0.28 (13.92–14.48)	– – –	– – –	ALL	CD
Michels et al. (2021)	ATFL CFL PTFL	13.20 ± 1.50 24.60 ± 2.30 –	10.40 ± 2.00 6.00 ± 1.90 –	1.40 ± 0.20 1.50 ± 0.40 –	N	CD
Milner and Soames (1998)	ATFL CFL PTFL	13.10 ± 3.90 (9.20–17.00) – –	11.00 ± 3.30 (7.70–14.30) – –	– – –	P,D,I	CD
Mun et al. (2020)	ATFL CFL PTFL	– – –	– – –	2.30 ± 0.60 (1.70–2.90) – –	NI	MRI
Neuschwander et al. (2013)	ATFL CFL PTFL	15.50–20.90 24.80 ± 2.40 (22.40–27.20) –	– – –	– – –	NI	CD
Peng et al. (2023)	ATFL CFL PTFL	– – –	– – –	1.88 ± 0.07 (1.81–1.95) – –	NI	MRI
Raheem and O'Brien (2011)	ATFL CFL PTFL	10.00–25.00 14.00–23.00 –	5.00–15.00 5.00–10.00 –	– – –	P,D,N	CD
Robbins et al. (2024)	ATFL CFL PTFL	9.30 ± 4.50 19.40 ± 3.70 –	– – –	– – –	N	CD
Rochelle et al. (2020)	ATFL CFL PTFL	12.60 ± 0.90 20.00 ± 1.90 13.40 ± 3.20	– – –	– – –	U	CD
Ruth (1961)	ATFL CFL PTFL	12.00 – –	5.00 6.00 –	– – –	U	MRI
Sarrafian (1993)	ATFL CFL PTFL	12.00 – 20.00 20.00–40.00 30.00	5.00–8.00 4.00–8.00 5.00	– – –	NI	U
Shi et al. (2024)	ATFL CFL PTFL	17.00 ± 0.80 – –	– – –	2.30 ± 0.40 – –	P,I	US
Siegler et al. (1988)	ATFL CFL PTFL	17.81 ± 3.05 (14.76–20.86) 27.00 ± 3.30 (24.70–30.30) 21.00 ± 3.86 (17.14–24.86)	– – –	– – –	NI	CD
Sindel et al. (1998)	ATFL CFL PTFL	12.58 – 21.38 26.80 ± 4.91 41.00 ± 2.81	3.41–7.76 6.00 ± 0.80 6.10 ± 0.77	– – –	NI	CD
Szaro et al. (2020)	ATFL CFL PTFL	16.10 – 32.80 24.50–42.70 –	5.80 7.90 –	1.30–2.90 1.30–2.50 –	NI	MRI
Szaro et al. (2021)	ATFL CFL PTFL	21.50 ± 0.50 (21.00–22.00) 27.50 ± 0.50 (27.00–28.00) –	5.00 ± 0.70 (4.30–5.70) 5.60 ± 0.30 (5.30–5.90) –	2.20 ± 0.05 (2.15–2.25) 2.10 ± 0.04 (2.06–2.14) –	NI	MRI
Taser et al. (2006)	ATFL CFL PTFL	22.37 ± 2.50 (19.87–24.87) 31.94 ± 3.68 (28.26–35.62) 21.66 ± 4.84 (16.82–26.50)	3.86–13.34 3.34–11.41 5.55 ± 1.25 (4.30–6.80)	– – –	N	CD
Teramoto et al. (2020)	ATFL CFL PTFL	– – –	4.00 ± 1.00 (3.00–5.00) 4.80 ± 0.60 (4.20–5.40) –	– – –	N	MRI
Testut and Latarjet (1948)	ATFL CFL PTFL	– 30.00–40.00 –	– 4.00–5.00 –	– – –	U	U
Uğurlu et al. (2010)	ATFL CFL PTFL	14.38–20.84 26.67 24.12	4.05–7.61 4.57 5.09	– – –	N	CD
Vega et al. (2018)	ATFL CFL PTFL	5.40–22.50 11.70–28.70 –	2.70–14.80 – –	– – –	P,D	CD
Wenny et al. (2015)	ATFL CFL PTFL	9.13 – 15.49 13.83–19.72 18.16–24.29	4.99–8.01 3.74–6.09 5.02–9.34	– – –	P,D,I	CD
Yabka and Bahia (2021)	ATFL CFL PTFL	– 26.85 ± 3.46 (23.39–30.31) –	– 5.33 ± 1.45 (3.88–6.78) –	– – –	N	CD
Yang et al. (2021)	ATFL CFL PTFL	9.35–21.66 – –	2.44–10.93 – –	0.42–0.96 – –	N	CD
Yildiz and Yalcin (2013)	ATFL CFL PTFL	10.25–16.21 12.09–23.01 –	11.07 ± 5.63 (5.44–16.70) 5.44 ± 2.34 (3.10–7.78) –	– – –	NI	CD
Yoon et al. (2018)	ATFL CFL PTFL	– – –	– – –	2.11–5.66 – –	NI	MRI
Yoshizuka et al. (2018)	ATFL CFL PTFL	– 17.70 ± 3.50 (14.20–21.20) –	– 3.90–11.00 –	– – –	N	CD

*Note*: The displayed results vary based on the different methods of reporting used in these studies.

Abbreviations: Ankle position: ALL, all movements of the ankle complex; CD,cadaveric dissection; D, dorsiflexion; I, inversion; Lig, ligaments; mm, millimetres; MRI, magnetic resonance imaging; N, neutral; NI, not indicated; P, plantarflexion; Study type: U, unknown; US, ultrasound.

Indeed, on closer examination of existing methodological approaches, it is apparent that there are inconsistencies in the characterization of full ligament length. The majority of literature considers full ligament length as the distance between the most proximal portion of the proximal attachment point to the most distal portion of the distal attachment point (Ahmed et al., 2024; Khawaji, 2016; Khawaji & Soames, 2015; Kitsoulis et al., 2011; Kobayashi et al., 2020; Kristen et al., 2019; Liu et al., 2017; Neuschwander et al., 2013; Siegler et al., 1988; Szaro et al., 2020, 2021; Yang et al., 2021; Yildiz & Yalcin, 2013; Yoon et al., 2018). However, some studies identify full ligament length as the region between attachment points, also known as the free ligament length (Milner & Soames, 1998; Taser et al., 2006; Wenny et al., 2015). This characterization fully disregards the entheses and attachment lengths of the ligaments, resulting in a reduced full‐ligament length measurement. The variability observed in measurements can be attributed, at least in part, to the differences in defining the complete length of a ligament.

Another area of inconsistency lies in the technique used to measure each ligament. Ligaments possess a limited presence of elastin (~5% of dry weight) which facilitates their capacity for controlled stretching and recoil (allowing for ~6% variation in length) (Benjamin et al., 2006; Henninger et al., 2015). Despite extensive literature available on the dimensions of the lateral ankle ligaments, existing studies overlook the dynamic nature of a ligament during full‐length measurement. Current methods for assessing the full length of the lateral ligaments involve measuring the distance between ligament attachment points. This produces a linear measurement which is effective for measuring a ligament under tension. This method, however, fails to account for the undulations or curvatures inherent in the relaxed ligament during movements of the ankle complex. As a result, there is significant variability among results between the measurements of a ligament under tension and a ligament in a relaxed state.

**FIGURE 1:**
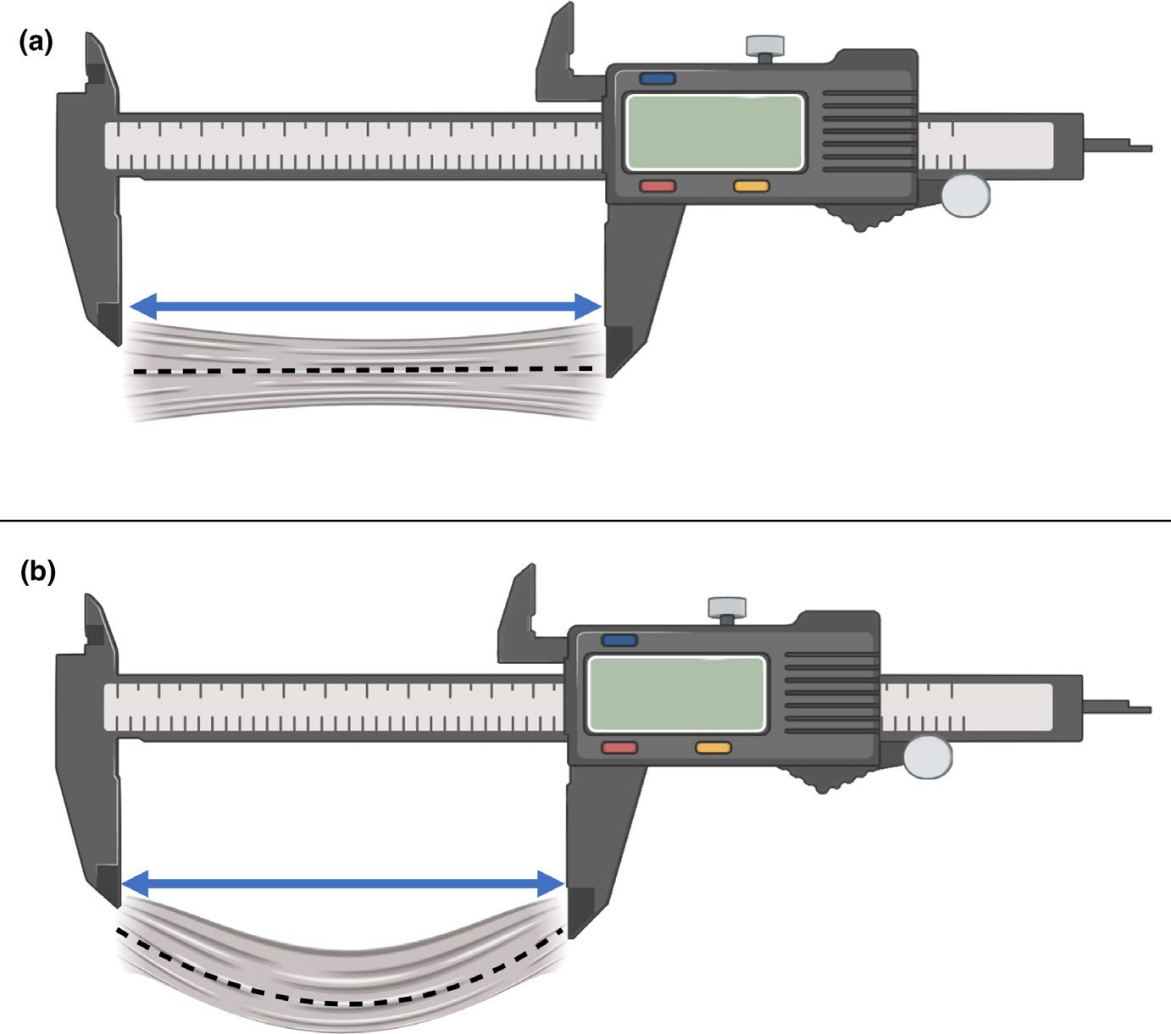
Measuring ligament length in different orientations. (a) When the ligament is taut the full ligament length can be measured. (b) However, when the ligament is relaxed the ligament may become folded and its full length is not easily measured. Measuring the distance between attachment points using inflexible tools does not take the form and degree of tension of the ligament into consideration. Blue arrow: Measured length between attachment points. Black dotted line: Indicates the curvatures of the ligament in maximum and minimum tension.

The lack of consideration for the flexible nature of the ligament during dimension assessment is further echoed in the instruments and measurement devices used. Instruments that lack the flexibility to adapt to curved surfaces and primarily focus on linear measurements are commonly used and mainly include either a calliper (Ahmed et al., 2024; Bai et al., 2013; Burks & Morgan, 1994; Edama et al., 2018; Inchai et al., 2023; Khawaji, 2016; Khawaji & Soames, 2015; Kobayashi et al., 2020; Milner & Soames, 1998; Neuschwander et al., 2013; Raheem & O'Brien, 2011; Taser et al., 2006; Uğurlu et al., 2010; Wenny et al., 2015; Yang et al., 2021; Yildiz & Yalcin, 2013), ruler (Wenny et al., 2015; Yabka & Bahia, 2021; Yoshizuka et al., 2018), flexible ruler (Kitsoulis et al., 2011), isometer (Buzzi et al., 1993), 3D coordinate measuring device (Clanton et al., 2014), ultrasound (Croy et al., 2012; Kawabata et al., 2023; Kristen et al., 2019; Peng et al., 2023), magnetic tracking device (Luo et al., 1997), and/or magnetic resonance imaging (de Asla et al., 2009; Dimmick et al., 2008; Liu et al., 2017; Mun et al., 2020; Szaro et al., 2020, 2021; Teramoto et al., 2020; Yoon et al., 2018). These instruments, while suitable for measuring the ligament under tension (between proximal and distal attachment points) and under mild relaxation (flexible ruler), lack the ability to fully conform and account for the degree of tension or laxity within the ligament during movement (Figure 1). Consequently, when a ligament is under tension, it is often perceived as ‘longer,’ whereas it is considered ‘shorter’ when relaxed (Khawaji, 2016). Despite the wealth of literature available on the dimensions of the lateral ankle ligaments, no study to date has accurately considered the flexible nature and possible undulating form of these ligaments during full‐length measurements. A measuring device which fully conforms to the ligament surface from its most proximal point to its most distal is therefore necessary to decipher the true full‐ligament length.

### 1.1 Study aims

In an effort to improve surgical outcomes and tissue‐engineered design, an accurate understanding of the dimensions of the lateral ligaments of the ankle is essential. Therefore, it is imperative to design a reliable and robust full‐length measurement method that considers the flexible form of the ligament throughout all movements of the ankle.

This study aims to address this gap through the development and validation of a new methodology known as the string for dynamic tissue (SDT) method. This method uses a combination of string and a digital calliper to accurately measure the full length of the lateral ankle ligaments, accounting for ligament curvature during all ankle movements. The flexible, pliable nature of the string enables the flexible form of the ligament to be closely followed, even when the ligament is in a relaxed state. The SDT method is implemented to assess thew dimensions of the lateral ankle ligaments and subsequently compares these measurements with those reported in existing literature. Through the consideration of the ligament form, this study hypothesizes that using the SDT method will yield greater values of relaxed ligament length when compared to the measurements (of the relaxed ligament) documented across existing literature. However, measurements of the ligament under maximal tension (taut) should remain similar to the results previously identified due to its linear state.

## 2 METHOD

### 2.1 Dissection and observation of the lateral ankle ligaments

Human cadaveric tissue was obtained from The University of Edinburgh Medical School body donation programme, regulated by The Anatomy Act (1984) and The Human Tissue (Scotland) Act (2006). All donors had previously consented to photography and all figures have been approved by the ethical review board (ethical review number: ANATED_0015). The lateral ligaments (ATFL, CFL and PTFL) of 21 ankles (12 female, 9 male, average age: 83 years, range: 62–96) were assessed from an initial pool of 12 pairs. Two injuries to ATFL and one injury to CFL were identified and were therefore not suitable to conduct measurements on. In one specimen, damage occurred solely to the ATFL during dissection, leaving the CFL and PTFL intact and uninjured. Consequently, this specimen was deemed suitable for assessing the CFL and PTFL only. This resulted in the assessment of 21 PTFL, 21 CFL, and 20 ATFL.

Fresh‐frozen cadaveric feet and ankles were thawed at 4°C for 48 h prior to each dissection. Each specimen was prepared and detached in a transverse plane at the lower third of the leg to preserve the talocrural and subtalar joints. The skin, fascia and surrounding musculature were removed from the anterior, posterior, medial and lateral portions of the lower leg and ankle and the dorsum and plantar aspect of the foot down to the midtarsal joint. The tendons of the extensor hallucis longus, extensor digitorum longus, tibialis anterior, peroneus longus, brevis and tertius, the calcaneal tendon, tendon of the tibialis posterior, flexor hallucis longus, and flexor digitorum longus muscles were sectioned and reflected to expose the ATFL, CFL, and PTFL. Fat and fascia located between each of the ligament bands and individual fibres and fibre bundles were carefully removed to fully expose the free length and entheses of each ligament. Once visible, the ligaments were assessed for intactness/injury, band number, and type. A full understanding of the morphometrics of the lateral ligaments of the ankle is essential to mimic the native structure in tissue‐engineered design. Multiband ligaments were identified when distinct sets of fibres exhibited separate points of origin or insertions. The tibia was dislocated from the talocrural joint in order to expose the PTFL.

### 2.2 Measurements of the lateral ankle ligaments

**FIGURE 2:**
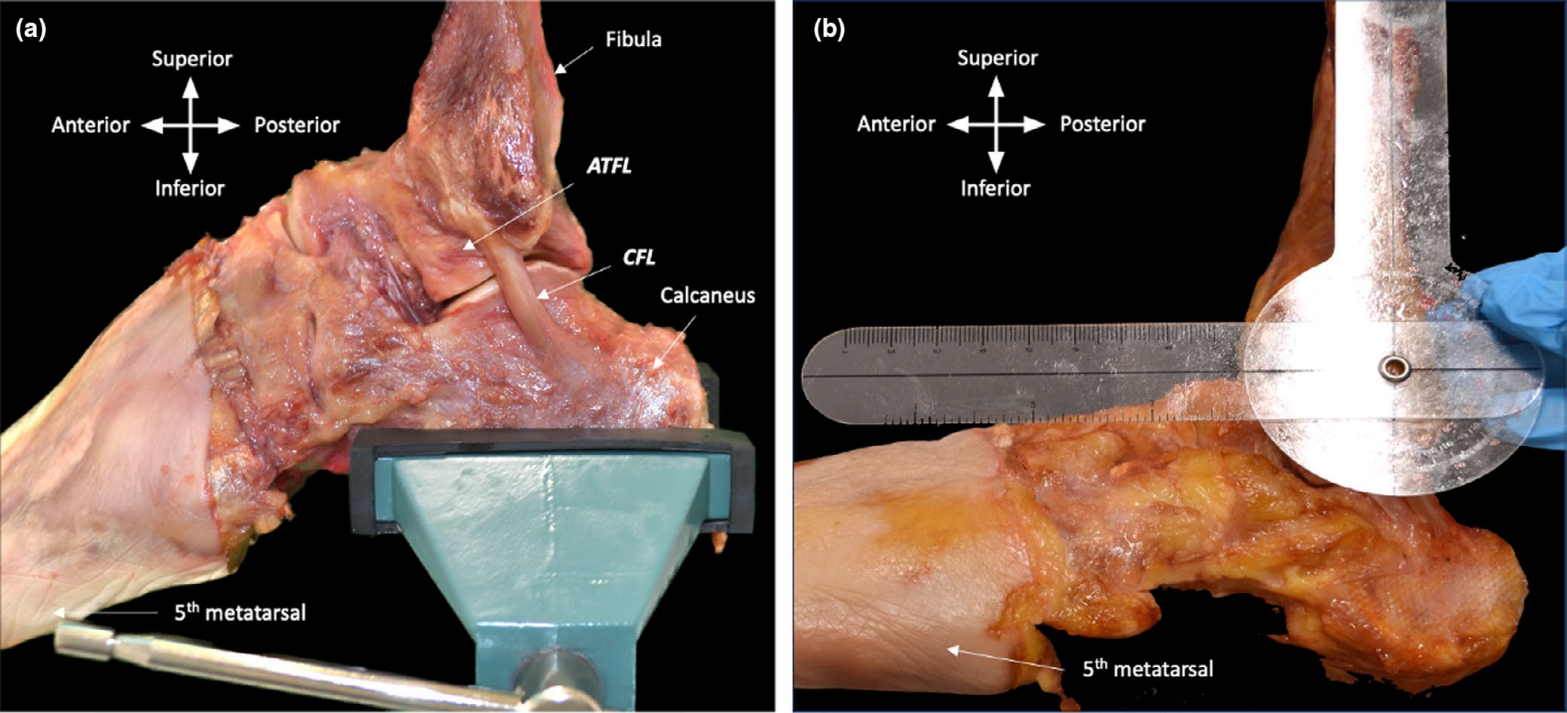
Positioning of the ankle complex. (a) Left ankle, skin and fascia removed to expose ATFL and CFL. Calcaneus clamped with vice to stabilize the ankle complex. (b) Left ankle, goniometer placed parallel to the fibula and shaft of the 5th metatarsal in neutral (90°).

The calcaneal tuberosity of the calcaneus was clamped with a vice to stabilize the talocrural and subtalar joint throughout measurements. Ankle complex movements were manually manipulated and held in place to ensure precision during measurement procedures. The passive range of motion of plantarflexion and dorsiflexion of each talocrural joint was determined using a goniometer (NCD medical, Prestige Medical). The fulcrum of the goniometer was positioned over the lateral malleolus. The stationary limb was aligned with the lateral aspect of the fibula, while the movable limb was set parallel to the 5th metatarsal, following the method described by Khawaji (2016) (Figure 2).

The starting position was the neutral position when the talocrural joint was positioned at a 90‐degree angle. Maximal plantar‐ and dorsiflexion were determined by their maximum range of movement in isolation from rotation of the subtalar joint. Therefore, the endpoint of plantarflexion was determined by the initial signs of internal rotation of the fibula and endpoint dorsiflexion by external rotation of the fibula.

**FIGURE 3:**
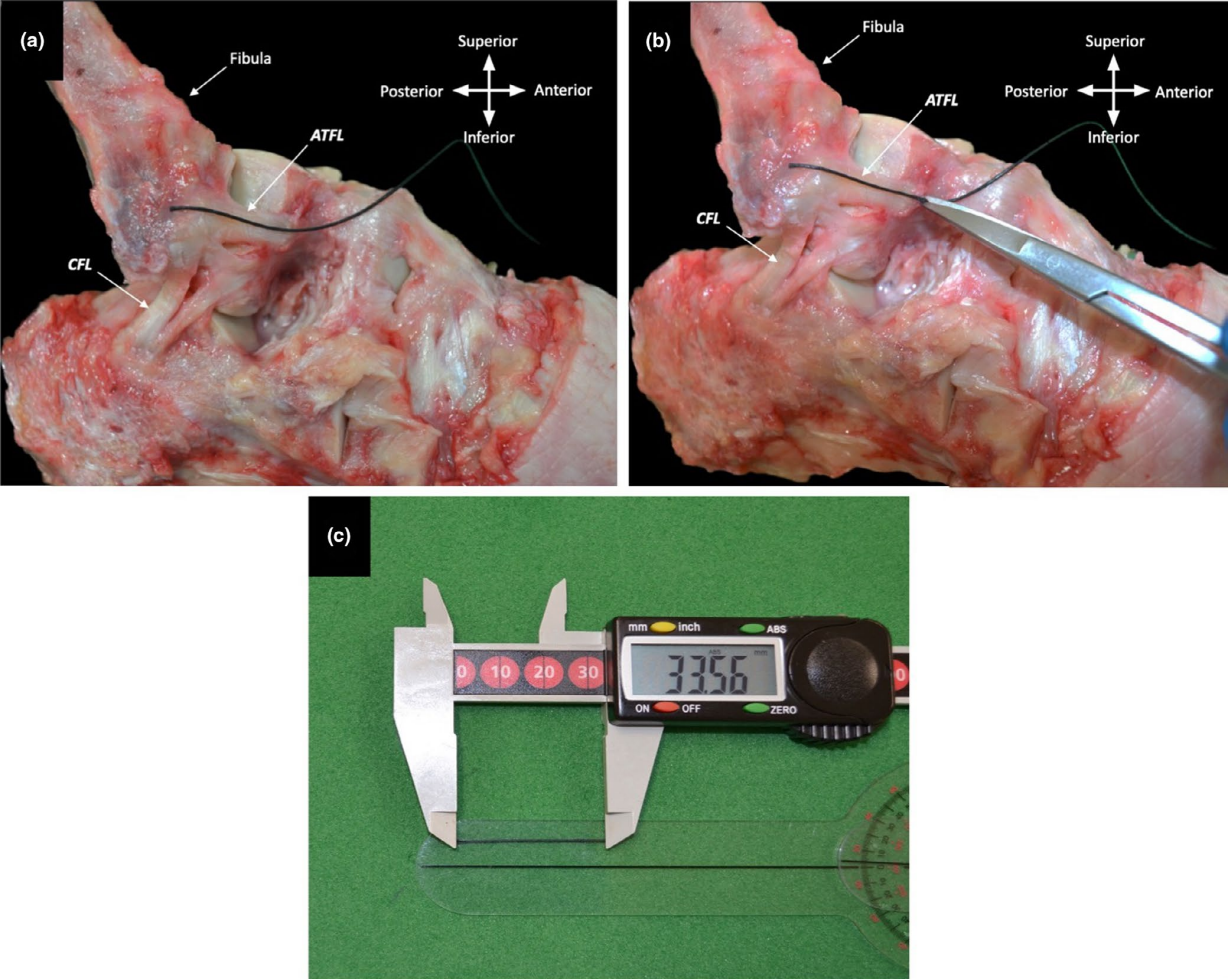
Full ligament length of ATFL, CFL, and PTFL. Proximal and distal attachment, full and free lengths of the lateral ligaments of the ankle. (a) Measurement regions for the ATFL. (b) Measurement regions for the CFL. (c) Measurement regions for the PTFL.

In line with the majority of current literature, full‐ligament length was quantified as the region between the most proximal point of the proximal attachment to the most distal point of the distal attachment point along the same plane. This equates to the combined measurements of its proximal and distal attachment lengths, along with the free ligament length (Figure 3). The free length of the ligament forms its ‘flexible’ portion.

Ligament length was determined with the ankle joint complex (talocrural and subtalar joint) in plantarflexion, dorsiflexion, inversion, eversion, and neutral to assess true ligament length throughout all movements.

### 2.3 The string for dynamic tissue (SDT) method

The SDT method uses a combination of string and a digital calliper to assess the full ligament length of the lateral ligaments of the ankle. Due to its mouldable and pliable properties, flax‐coated wax string (Barbour, flax linen thread with wax 18/5 ply, Leatherhouse) was used to measure the full ligament length. Flax‐coated wax string is robust yet has sufficient flexibility to fully conform to the ligamentous surface even in instances where it folds over on itself. The wax coating of the string allowed the string to be easily marked with a blade to demonstrate the exact length of each ligament. It is therefore a highly suitable tool for assessing full ligament length.

**FIGURE 4:**
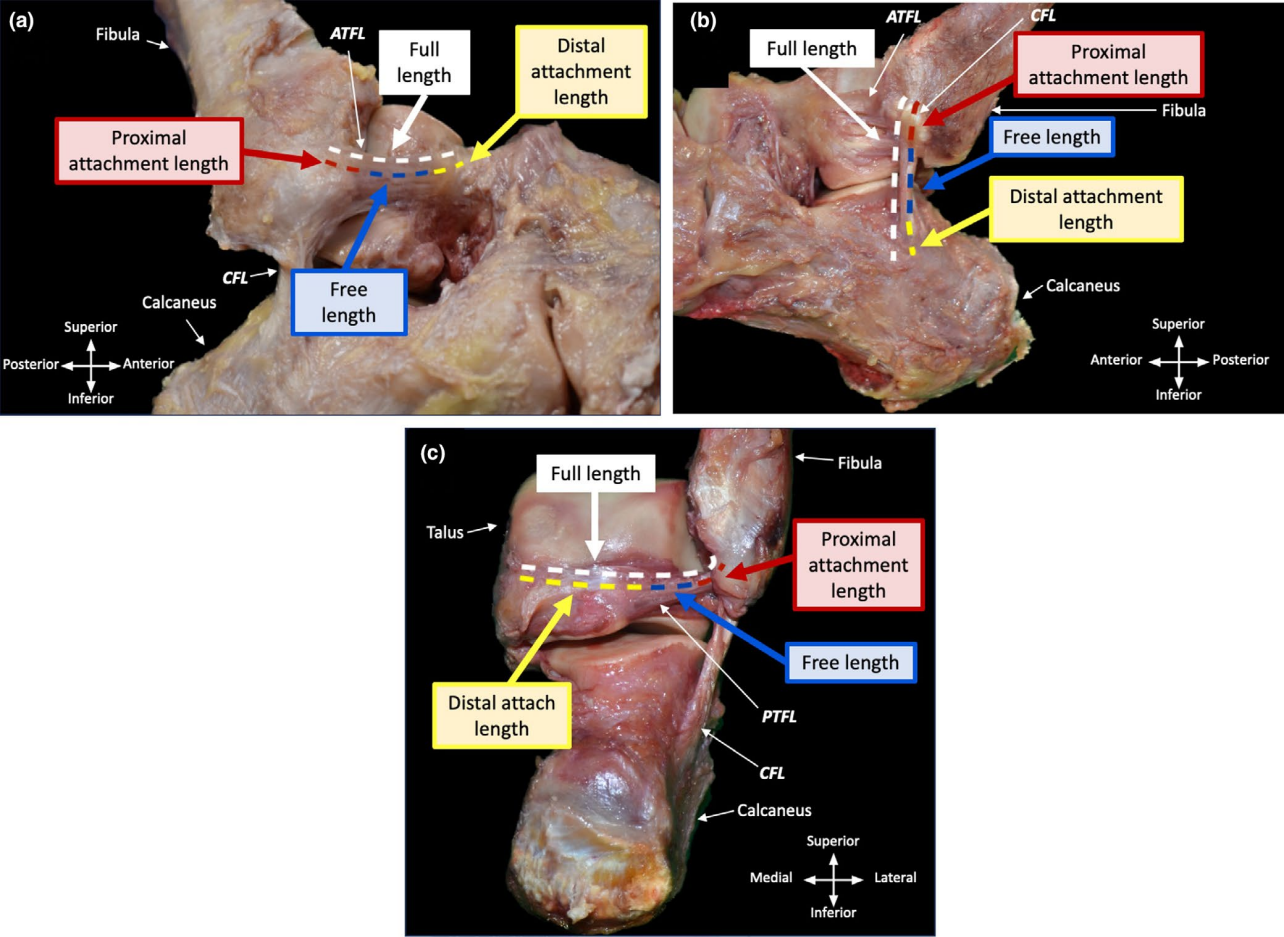
The SDT method. (a) Flax‐coated string aligned to the superior band of ATFL in passive dorsiflexion placed at the most proximal part of the proximal enthesis of ATFL. (b) An indentation is made on the flax‐coated string where the ligament ends using curved dissection scissors. The flax‐coated string is cut to size to match the exact length of the ligament. (c) The string is flattened and straightened and then measured using a digital calliper, and the length of the string and subsequent ligament is recorded.

Once the ankle joint complex (talocrural and subtalar joint) was set into the correct position and held, the string was aligned to the ligament from the most proximal portion of the ligament along its entire length on the same plane (Figure 4). At its most distal point, an indentation to the wax‐coated string was made using a set of curved dissection scissors to mark the exact end point of the ligament. The string was then removed from the ligament and cut to size at its indentation. The cut string was smoothed out and placed under a coverslip. Lightweight pressure was applied to ensure the string lay uniformly flat and achieved full extension. A digital calliper (Baty BAT‐DC‐1‐WP, Sussex, England, accuracy: ±0.02 mm) was then used to measure the exact length of the string and subsequently the true length of the ligament. This technique was repeated on all the lateral ligaments of the ankle through all movements of the ankle and subtalar joint (plantarflexion, dorsiflexion, inversion, eversion, and neutral). In multiband ligaments, measurements were focused on the longest ligamentous band. Within the present study, the multiband structure was observed in the ATFL and PTFL, where the ligamentous bands often merged along its midportion and/or proximally and distally, thereby complicating the ability to measure them separately. When two bands were present, the longest band was the superior band of the ATFL and the posterior band of the PTFL.

### 2.4 Reliability of measurement methodology

Reliability was assessed using intraclass correlation coefficient (ICC) as recommended by Koo and Li (2016) using SPSS version 27 (IBM, Armonk, USA) statistical package. In order to assess the intra‐observer reliability of this methodology, three separate series of measurements were assessed (throughout all movements of the ankle complex) on two separate (unpaired) ankles. Reliability and repeatability were assessed for full ligament length (using string method), ligament width and thickness, and proximal and distal ligament width and length. The measurements were taken on separate days by the same observer (between 2 and 4 days apart).

Inter‐observer reliability of this methodology was assessed with two separate observers (one experienced anatomist and one non‐anatomist) on two separate (unpaired) ankles. Full ligament length was assessed throughout all movements of the ankle complex. A comprehensive description of the methodology was provided for each observer to follow. Using observers with a variety of experience opens the repeatability and reliability of this method to a range of users (both anatomists and non‐anatomists).

### 2.5 Statistical testing

To test the hypothesis that minimum ligament measurements (in a relaxed state) differ significantly from those reported in the literature, while maximum measurements (under tension) do not, a comparison of the average minimum and maximum values from both the literature and the current study would be required. However, due to inconsistencies in the literature, such as limited reporting of movement‐specific values, reliance on ranges rather than exact measurements, and variability in the number of movements studied, a direct statistical comparison is not feasible. Instead, the hypothesis will be assessed by contextualizing the results from this study within the broader patterns and trends observed in the literature, following guidance from a biomedical statistician.

## 3 RESULTS

### 3.1 Reliability

Minimal differences were observed in measurements taken by the same observer, with an average intra‐observer ICC of 0.997 (95% confidence interval: 0.995–0.998) across observations 1, 2, and 3. Similarly, minimal variation was noted between observers 1, 2, and 3, with an average inter‐observer ICC of 0.995 (95% confidence interval: 0.992–0.997). These findings indicate that the method is highly reliable, and the measurements obtained are consistent and repeatable.

### 3.2 General findings

The ATFL most commonly presented with two ligamentous bands extending between the distal fibula and talus (*n* = 16). The ATFL of two ankles presented with a single ligamentous band, while two other ankles displayed three ligamentous bands. The CFL typically presented as a flat ligamentous band (*n* = 17) as opposed to a cord‐like ligament structure (*n* = 4). A single ligamentous CFL was only identified throughout the sample (type I). PTFL presented as a trapezoidal‐shaped ligament with two ligamentous bundles: a longer posterior bundle and a shorter anterior bundle. In all samples, the inferior band of the ATFL (if present) was found to connect to the proximal aspect of the CFL through arciform fibres.

### 3.3 Ligament measurements

#### 3.3.1 ATFL

##### Length

The average length of the ATFL (*n* = 20) ranged from 25.89 ± 0.53 to 28.92 ± 0.46 mm, reflecting measurements across all positions of the ankle complex (Table 2). The ATFL was observed and identified to be under maximal tension, and therefore measuring its longest, during subtalar joint inversion and relaxed during dorsiflexion and eversion. There is only a minimal difference between the measurements of the ligament throughout all degrees of movement, with average measurements differing by ±3.03 mm (Table 2).

**TABLE 2:** Maximum, minimum, and mean (±SE) full‐length measurements of ATFL throughout all degrees of movement of the ankle and subtalar joint.

Movement	Min. Measurement (mm)	Max. Measurement (mm)	Mean (mm)
Max. plantarflexion	24.36	32.66	28.51 ± 0.55
Max. dorsiflexion	21.20	30.23	26.09 ± 0.54
Max. inversion	25.76	33.80	28.92 ± 0.46
Max. eversion	22.23	31.93	25.89 ± 0.53
Neutral	22.90	32.73	26.73 ± 0.53

#### 3.3.2 CFL

##### Length

The average length of the CFL ranged from 34.65 ± 0.76 to 37.10 ± 0.79 mm in length, based on measurements taken across all positions of the ankle complex (Table 3). The CFL was identified to be taut during inversion, followed by maximum dorsiflexion and was relaxed during subtalar joint eversion and ankle joint plantarflexion. The maximum difference between average measurements of the CFL in all movements is ±2.45 mm (Table 3).

**TABLE 3:** Minimum, maximum, and mean (±SE) full‐length measurements of the CFL throughout all degrees of movement of the ankle and subtalar joint.

Movement	Min. Measurement (mm)	Max. Measurement (mm)	Mean (mm)
Max. plantarflexion	28.75	42.54	34.69 ± 0.77
Max. dorsiflexion	32.97	43.79	36.79 ± 0.65
Max. inversion	30.20	44.44	37.10 ± 0.79
Max. eversion	28.66	42.24	34.65 ± 0.76
Neutral	32.07	42.01	35.41 ± 0.66

#### 3.3.3 PTFL

##### Length

The average length of the PTFL throughout all movements of the ankle complex ranged from 33.34 ± 0.55 to 35.27 ± 0.52 mm (Table 4). On average, the PTFL was identified to be under maximal tension during dorsiflexion and inversion and under minimal tension when the ankle is plantarflexed. All measurements of the PTFL only have minor differences, with maximum and minimum average measurements throughout all phases of movement differing by ±1.93 mm (Table 4).

**TABLE 4:** Minimum, maximum, and mean (±SE) full‐length measurements of the PTFL throughout all degrees of movement of the ankle and subtalar joint.

Movement	Min. Measurements (mm)	Max. Measurements (mm)	Mean (mm)
Max. plantarflexion	29.47	38.02	33.34 ± 0.55
Max. dorsiflexion	30.61	39.80	35.27 ± 0.52
Max. inversion	30.63	39.78	35.22 ± 0.52
Max. eversion	29.13	38.06	33.71 ± 0.54
Neutral	27.90	38.04	33.68 ± 0.60

#### 3.3.4 SDT method versus literature

On comparing the total average minimum and maximum measurements of the ATFL, CFL, and PTFL from this study with those reported in previous literature, a noticeable difference was observed for the minimum measurements. In contrast, no difference was found for the maximum measurements. These findings align with the initial hypothesis, demonstrating the reliability of this method (Table 5).

**TABLE 5:** Full ligament lengths of ATFL, CFL, and PTFL within the current study and throughout the literature.

	Current study		Other studies	
	Min. Measurement (mm)	Max. Measurement (mm)	Min. Measurement (mm)	Max. Measurement (mm)
ATFL	21.20	33.80	10.00^a^	32.80^b^
CFL	28.66	44.44	14.00^a^	42.70^b^
PTFL	27.90	39.80	13.92^c^	43.81^d^

^a^ Raheem and O'Brien (2011).

^b^ Szaro et al. (2020).

^c^ Luo et al. (1997).

^d^ Sindel et al. (1998).

## 4 DISCUSSION

High rates of injury and unsatisfactory treatment outcomes have established the lateral ligaments of the ankle as a focal point for extensive research. Numerous studies concentrate on exploring the anatomical structure of this ligament group, with the objective of refining surgical techniques and ultimately enhancing treatment outcomes. However, despite the abundance of literature surrounding the lateral ankle ligaments, treatment outcomes remain poor, and notable gaps remain in required knowledge for improved ligament healing.

Obtaining accurate dimensions of the lateral ligaments of the ankle can offer vital insights into both anatomic and non‐anatomic surgical repair and are a key element for the design of a successful tissue‐engineered replacement (Burks & Morgan, 1994; Park et al., 2021). To date, no study has considered the flexible form of the ankle ligaments during dimensional assessment and therefore full‐length measurements reported elsewhere fail to offer an accurate representation of true ligament length throughout all states of movement.

This study is the first to fully consider the dynamic and flexible nature of the lateral ligaments of the ankle during full‐length dimensional assessment. By devising and validating the SDT method, this study identified that in a relaxed state, the lateral ankle ligaments appear to exhibit a greater length than previously documented in the literature, supporting the initial hypothesis. This measured increase in relaxed ligament length can be attributed to the consideration of the undulated ligamentous surface. As mentioned previously, when a ligament is in a relaxed state, it may sag or even fold over onto itself, producing a wavy or undulating surface. Previous studies fail to consider the undulating ligament form, measuring only the distance between attachment points. Therefore, current reports of relaxed full ligament length are often misrepresented as being considerably shorter in length.

### 4.1 ATFL

The full ligament length of the ATFL ranged from 21.20 to 33.80 mm. This indicates an increased difference of 11.20 mm between the minimum measurements found in this study and minimum measurements found across the literature (Table 5). As predicted, maximum measurements of the ATFL mirrored maximal measurements identified within the literature, only differing by 1.00 mm on average (Table 5). Therefore, as hypothesized, the consideration of the curves and undulations of the relaxed ligament appears to have led to an increased measured length.

In agreement with other studies, the ATFL was identified to be under maximal tension during inversion and plantarflexion of the ankle complex and relaxed during dorsiflexion (Buzzi et al., 1993; Colville et al., 1990; de Asla et al., 2009; Edama et al., 2019; Hertel, 2002; Khawaji, 2016; Khawaji & Soames, 2015; Luo et al., 1997; Ozeki et al., 2006; Takeuchi et al., 2021). Ligaments are only functional when they are under tension (Solomonow, 2009). When a ligament is compressed or shortened/relaxed, it is non‐functional (Solomonow, 2009). Biomechanical injury typically arises when a ligament is subjected to tension. However, it is crucial to note that injury does not solely stem from tension alone (Lane & Amiel, 2017). Instead, it frequently manifests due to the cumulative effects of repetitive loading and strain experienced while under tension, leading to disruption of the ligament crimp pattern (Lane & Amiel, 2017). Therefore, this study confirms that the ATFL is at a higher risk of injury when the ankle is in an inverted and plantarflexed position (D'Hooghe et al., 2020; Fong et al., 2009; Kikumoto et al., 2019; Luo et al., 1997).

### 4.2 CFL

The CFL ranged from 28.66 to 44.44 mm, with an average full‐length measurement range of 34.65 ± 0.76–37.10 ± 0.65 mm. Due to the consideration of the ligament undulations during dimension assessment, minimum measurements of the relaxed ligament have shown an increase of 14.7 mm when compared to minimum measurements reported elsewhere (Table 5). As expected, maximum measurements of the CFL (measurement of the CFL under maximal tension) from this study and other studies were similar, only differing by 1.74 mm (Table 5). These findings strengthen the hypothesis and highlight the importance of considering the undulated surface of soft tissue structures during dimension assessment.

In agreement with Colville et al. (1990), Cawley and France (1991), Buzzi et al. (1993), Luo et al. (1997), Bahr et al. (1998), Hertel (2002), Ozeki et al. (2002), Sarrafian and Kelikian (2011), and Michels et al. (2023), the CFL was identified to be under maximal tension during inversion and dorsiflexion of the ankle complex and relaxed during maximal eversion and/or plantarflexion. While this is the most common finding within the current literature, previous studies have observed some differences in CFL tension throughout movements of the ankle complex. Previous research conducted by Khawaji (2016) and de Asla et al. (2009) yielded findings consistent with the present study, indicating that the CFL is under tension during ankle joint dorsiflexion and relaxed in plantarflexion. However, diverging from the present study, their findings also revealed increased CFL tension during subtalar joint eversion with reduced tension during subtalar joint inversion (de Asla et al., 2009; Khawaji, 2016). In contrast, Edama et al. (2017) identified increased CFL tension during inversion and plantarflexion when compared to inversion and dorsiflexion. Moreover, it has been observed that in some individuals, the tension of this ligament remains consistently taut throughout all movements of the ankle complex (Sarrafian & Kelikian, 2011). While existing literature most commonly characterizes the CFL as taut during dorsiflexion/inversion and relaxed on plantarflexion, some contradictions between results remain. This is thought to be due to differences between the angle of the CFL and the long axis of the fibula, otherwise known as the CFL running angle (Ozeki et al., 2002; Sarrafian & Kelikian, 2011). The CFL running angle has been identified to differ between individuals, resulting in varying degrees of ligament tension throughout movements of the ankle complex (Sarrafian & Kelikian, 2011). A CFL running angle <39° typically results in contraction during plantarflexion/eversion and stretching during dorsiflexion/inversion, while an increase to this angle (40°–59°) resulted in extension of the ligament during plantarflexion and contraction during dorsiflexion (Edama et al., 2017, 2019). While the CFL is most commonly reported to be under maximal tension during dorsiflexion of the ankle joint, Edama et al. (2017) suggest that a CFL running angle of 40° is most prevalent (35.8%), implying that the ligament is more likely to undergo stretching during plantarflexion and relaxation during dorsiflexion. This result, however, is not reflected within the current study or throughout the wider body of literature on this topic. Nevertheless, the second most frequently observed category, characterized by a CFL running angle of 30° which stimulates contraction of the CFL during plantarflexion and extension during dorsiflexion, was also prevalent, accounting for 27.2% of cases (Edama et al., 2017). All CFL's within the current study demonstrated elongation during dorsiflexion and therefore the results obtained within the current study fall within the CFL 30° category. Therefore, in line with previous research, this study confirms that the CFL is at higher risk of injury during subtalar joint inversion (Lapointe et al., 1997; Luo et al., 1997).

Previous literature highlights a potential anatomical connection between the inferior band of the ATFL and proximal CFL, suggesting a more integrated relationship between these structures in providing stability to the ankle joint (Cordier et al., 2021, 2023; Dalmau‐Pastor et al., 2020; Esparó et al., 2024; Golanó et al., 2010; Vega et al., 2018). Calliper measurements have shown that both ligaments maintain a constant length throughout ankle movements, supporting their classification as isometric structures (Vega et al., 2018). Their shared anatomical origin, connection via arciform fibres, and isometric properties have led to their classification as a single anatomical entity known as the lateral fibulotalocalcaneal ligament complex (LFTCL) (Vega et al., 2018). Isolated injury to the superior band of the ATFL has been described to result in microinstability, while involvement of the LFTCL is associated with chronic ankle instability (Basciani et al., 2024; Vega et al., 2018; Vega & Dalmau‐Pastor, 2023).

Consistent with previous research, arciform fibres were identified between the inferior band of the ATFL and the proximal aspect of the CFL in all double‐ and triple‐band structures within the current study (Dalmau‐Pastor et al., 2020; Golanó et al., 2010; Vega et al., 2018). However, using the SDT method and considering the curvatures of the relaxed ligament, the CFL was found to be longer under maximal tension and shorter when relaxed, indicating that the CFL is not an isometric structure. Instead, it operates in a manner similar to the superior band of the ATFL, dynamically contributing to the stability of the ankle complex. While this suggests that the CFL may not function in the same manner as the inferior band of the ATFL, it does not diminish its role as a component of the LFTCL. The CFL still originates from a similar location and is structurally linked to the inferior band of the ATFL, highlighting the close relationship between these two ligament fascicles.

### 4.3 PTFL

Full ligament lengths of the PTFL ranged from 27.90 to 39.80 mm, with average measurements ranging from 33.34 ± 0.55 to 35.27 ± 0.52 mm. By incorporating the curvatures of the PTFL in dimension assessment, the full length of the contracted ligament observed in the present study exceeded previous measurements by 13.98 mm (Table 5), thereby highlighting the importance of considering ligament form in achieving accurate and precise results in the area of ligament measurement. Maximum measurements of the PTFL (measurements of the taut ligament) were similar to measurements reported elsewhere, only differing by 4.01 mm (Table 5) in length. These findings reinforce the hypothesis and highlight the importance of accounting for the undulated surface of soft tissue structures when assessing their dimensions.

As reflected in the current literature, the PTFL is identified to be under maximal tension and therefore measuring its longest during maximal dorsiflexion and relaxed during the neutral position and maximal eversion (Buzzi et al., 1993; Colville et al., 1990; Luo et al., 1997; Ozeki et al., 2006). This study confirms that the PTFL is at a higher risk of injury during maximal dorsiflexion of the ankle joint.

Consideration of the curved surface of the ATFL, CFL, and PTFL leads to both individual and overall increased measurement values when compared with the current literature. The SDT method has therefore proven to be a more representative and anatomically precise technique for assessing dynamic curved soft tissue structures. Therefore, as hypothesized, the consideration of the curves and undulations of the relaxed ligament appears to have led to an increased measured length, though this finding has not been statistically tested.

### 4.4 Applications of the SDT method

The SDT method effectively highlights the significance of considering ligamentous form during dimension assessment throughout all movements of the ankle complex. It enables the precise evaluation of full ligament length, accounting for both taut and relaxed states, offering accurate anatomical guidance for surgical management and the development of tissue‐engineered replacements. Whether performing a Broström repair, involving ligament tightening, or reinforcing with a tendon graft, precise knowledge of the lateral ankle ligament's dimensions is imperative (Wenny et al., 2015). Understanding the exact length of the ligament enables surgeons to determine the appropriate degree of tightening required or the necessary tendon length for reinforcement in order to return the ligament to its normal function (Wenny et al., 2015). Further to this, changes in ligament dimensions can frequently signify injury or trauma as a result of inflammation (Hayes et al., 2019). Therefore, a thorough knowledge of the dimensions of the native tissue enhances the overall accuracy of treatment design and diagnosis, ultimately contributing to improved patient outcomes. Accurate dimensions of the native tissue also play an integral role in ensuring the tissue‐engineered ligament has the same contraction and recoil abilities as the native tissue (Loukopoulou et al., 2022). Establishing minimum and maximum measurements helps guide the development process, allowing the resulting structure to expand to its full extent and recoil to its smallest size, mimicking the natural behaviour of the native tissue.

Flexible instruments such as the flexible ruler have been used successfully to measure curvatures in both soft tissue and bone throughout the human body. Although these instruments work well for mapping gentle curves like those in the spine (Hart & Rose, 1986; Rheault et al., 1989; Simpson, 1989), bony contours (Zeng et al., 2023), and connective tissue surfaces (Ge et al., 2020; Paul et al., 2011), they lack the ability to fully conform to the sharp angles of a relaxed ligament. Unlike the flexible ruler, the SDT method possesses the versatility and flexibility to fully conform to any curved or nonlinear surface. This method is therefore not confined solely to ligamentous tissue but can be used to assess the dimensions of any given structure, such as tendons, cartilage, or even curvatures of the clavicle or rib bones.

Further to this, the SDT method may also aid in determining connective tissue slack length. Numerous studies have examined the concept of “slack length” in muscles, tendons, and ligaments; however, this term lacks a consistent definition across the literature. Some researchers define slack length as the relaxed length of a soft tissue structure (Baxter et al., 2019; Farshidfar et al., 2022), while others describe it as the length at which a connective tissue structure starts to exhibit initial tension or loading (Bloemker et al., 2012; Stubbs et al., 2018; Zhang et al., 2021). Regardless of its definition, slack length is commonly measured in vivo through the evaluation of the degree of tension or strain within a soft tissue structure (Zhang et al., 2021). The three most common methods for measuring strain are: implantable strain sensors, the virtual fibre elongation technique, and ultrasonic or shear imaging (Hug et al., 2013; Zhang et al., 2021). While these approaches have had some success in assessing in vivo strain, they each present specific challenges when it comes to accurately measuring slack length or determining full ligament length throughout all movements of the ankle complex (Hug et al., 2013, Zhang et al., 2021). Although the SDT method does not have the ability to measure strain or tension, it can provide accurate full‐length measurements of a ligament. Full ligament length is an essential component in the formulation of slacklength; therefore, suggesting that the SDT method may also be useful in this context (Manal & Buchanan, 2004). This highly versatile SDT methodology removes the need for expensive equipment or complex software and uses affordable and accessible tools to accurately assess the dimensions of any given anatomical structure.

### 4.5 Limitations

This study has several limitations. The sample size for this study was limited due to the use of cadaveric specimens. While this sample size is in line with surrounding literature in this field (ranging between 8 and 50 samples), a reduced sample size may lead to findings that inadequately capture the diversity of the broader population or lack generalizability across all demographic groups.

An additional limitation of the sample lies in the use of paired right and left ankle specimens from the same individuals, with multiple ligaments derived from the same ankle. This introduces non‐independence in the data, which is an important consideration despite not being statistically tested.

A further limitation of the method developed in this study is that it is restricted to cadaveric specimens, thereby making it a post‐mortem technique, reducing its potential for in vivo applications.

Similarly, the utilization of cadaveric specimens presents limitations in accurately representing human subjects, attributed to the absence of weight‐bearing biomechanics and dynamic physiological processes. As a result of the COVID‐19 pandemic, the body donation programme at the University of Edinburgh, Scotland was closed between March 2020 and September 2021, resulting in a decrease in donor availability (Brassett et al., 2020). The cadavers used for this study had an age range of 62–96 years; inclusion of a more diverse range of age groups may have resulted in a greater range of motion identified within the ankle complex and therefore a greater variety of ligament dimensions. Previous research by Grimston et al. (1993) and Nigg et al. (1994) has identified a reduction in ankle range of motion with increasing age, thereby emphasizing the importance of incorporating diverse age groups when evaluating ligament length. Another area of limitation within this study was the lack of in‐depth cadaveric medical history. A complete history of pathologies affecting the lower limb and foot may aid in developing our understanding of presenting variety between ligament dimensions. Another limitation was the inability to perform statistical testing to compare the measurements from this study with those reported in the literature, due to inconsistencies in data reporting across sources. To properly assess the hypothesis, a larger‐scale study with standardized data parameters should be conducted, enabling a more accurate statistical comparison of the results.

Finally, the range of motion and biomechanical alterations observed in the frozen–thawed cadaveric ankle may vary from those documented during weight‐bearing movement in the ankle complex, as previously observed in frozen–thawed posterior tibial tendons (Giannini et al., 2008). Therefore, the results obtained from this study may not directly translate to the weight‐bearing ankle complex in vivo. While fresh‐frozen cadaveric ankle specimens differ from weight‐bearing ankles in this regard, they remain preferable to embalmed specimens (Hohmann et al., 2019). Previous studies have reported minimal biomechanical changes in frozen–thawed specimens and no loss of structural integrity in tendons and ligaments (Arnout et al., 2013; Bell et al., 2020; Hohmann et al., 2019; Jansen et al., 2020). However, some alterations in soft tissue stiffness and joint laxity may still occur, which could introduce variation in biomechanical testing. While the extent of this effect remains unclear, prior research suggests it is unlikely to have had a significant impact on the overall findings (Arnout et al., 2013, Bell et al., 2020, Hohmann et al., 2019, Jansen et al., 2020).

## 5 CONCLUSION

This study is the first of its kind to consider the form and dynamic, flexible nature of the lateral ankle ligaments throughout dimensional assessment. The development and application of the SDT method were successful in accurately measuring the full ligament length of the ATFL, CFL, and PTFL in both a relaxed and taut state. This new methodology proved highly repeatable and reliable across users. As hypothesized, consideration of the form and undulations of the relaxed ligament during dimension assessment resulted in increased full‐length measurements when compared to similar measurements within the current literature. This new methodology provides precise and accurate full‐length measurements of the lateral ligaments of the ankle to guide surgical repair and to establish the creation of tissue‐engineered replacements for lateral ankle ligament replacement.

## AUTHOR CONTRIBUTIONS

Concept/design: J.Z.P. and S.J.M., acquisition of data, data analysis/interpretation, drafting of the manuscript: S.J.M., critical revision of the manuscript and approval of the article: J.Z.P. and A.H.R.W.S.

## Data Availability

The data that support the findings of this study are available from the corresponding author upon reasonable request.
